# Dynamical Projective Operatorial Approach (DPOA): Theory and Applications to Pump–Probe Setups and Semiconductors

**DOI:** 10.3390/ma18061310

**Published:** 2025-03-16

**Authors:** Amir Eskandari-asl, Adolfo Avella

**Affiliations:** 1 Dipartimento di Fisica “E.R. Caianiello”, Università degli Studi di Salerno, I-84084 Fisciano, SA, Italy; aeskandariasl@unisa.it; 2 CNR-SPIN c/o Università degli Studi di Salerno, I-84084 Fisciano, SA, Italy; 3 CNISM c/o Università degli Studi di Salerno, I-84084 Fisciano, SA, Italy

**Keywords:** pump–probe setups, semiconductors, dynamical projective operatorial approach (DPOA)

## Abstract

This manuscript reviews our recently developed theory, the dynamical projective operatorial approach (DPOA), for studying pump–probe setups in ultra-fast regimes. After reviewing the general formulation of the DPOA, we focus on its lattice version and provide a formalism that is particularly suitable for several pumped semiconductors. Within the DPOA, we also compute the TR-ARPES signal through out-of-equilibrium Green’s functions and establish an out-of-equilibrium counterpart of the fluctuation–dissipation theorem. Moreover, we generalize the linear response theory to pumped systems and address, within the DPOA, the differential transient optical properties, providing an overall robust theoretical and computational framework for studying pump–probe setups. Considering a minimal model for a semiconductor, we illustrate the capabilities of the DPOA and discuss several features emerging in this case study that are relevant to real materials.

## 1. Introduction

In the recent few decades, by the so-called pump–probe setups being developed, it became possible to unravel out-of-equilibrium particle dynamics in femtosecond and even sub-femtosecond time regimes [1,2,3,4,5,6,7,8]. The importance of these studies is two-fold. On the application side, gaining control over systems in such short time scales paves the way towards ultra-fast electronics. On the fundamental physics side, the real-time dynamics of pump-induced charge excitations determines the underlying physical mechanisms [9,10,11,12,13].

The pump pulse is usually in the range of energy gaps between the valence band (VB) and conduction band (CB), which would be in the IR regime for typical semiconductors. Pumping the system excites electrons and changes their energy distributions, which is a crucial subject of investigation. In general, there are three approaches to reading out the response of a system to an ultra-short, intense pump pulse. One approach is to study the high-harmonic generation induced by the pump pulse [14,15,16,17,18,19,20,21,22,23], which is not discussed in this manuscript. Another important approach is to study the time-resolved angle-resolved photoemission spectroscopy (TR-ARPES) signal [13,24,25,26,27,28,29,30,31], which is an out-of-equilibrium version of ARPES [32,33,34,35,36,37,38]. It uses a probe pulse to eject electrons from the sample and analyzes their energy and momentum distribution. These distributions contain information about out-of-equilibrium bands and their occupations and vary depending on the time delay between pump and probe pulses.

The third and last approach is centered around studying the transient optical response of the pumped system to a very low-intensity probe pulse [9,10,20,39,40,41,42,43,44,45,46,47,48,49,50,51,52,53,54]. The most convenient probe pulse has a high frequency, which is used to excite electrons from the core bands to the VBs and CBs near the Fermi surface. The pump pulse excites electrons and holes in these CBs and VBs, while the probe pulse reveals the details of these excitations through exciting core-band electrons to them, which are readable from the optical response of the system to the probe pulse.

The aforementioned experimental advances demand the development of appropriate theoretical tools to both simulate and analyze the experimental results. The most standard approach for this aim is the time-dependent density functional theory (TD-DFT) [47,54,55,56,57,58,59,60,61]. Despite several advantages of TD-DFT, it unfortunately has two significant disadvantages. First, the computational cost is very high, as it demands considerable resources, and the calculations usually take very long to compute [62]. Second, it is not very easy to gain deep insights into the individual roles of different mechanisms and understand how they interplay with each other [62].

Alongside the *ab initio* approaches, there are model-Hamiltonian approaches that can be used for the pump–probe setups [47,63,64,65], with parameters that are usually obtained from equilibrium DFT calculations [66]. These approaches straightforwardly solve the second problem of TD-DFT, as one can turn on and off different terms of the governing Hamiltonian to see how the final results are affected. However, it is generally challenging to handle the numerous complications of *real* setups within model-Hamiltonian approaches, and this may result in oversimplification [62].

To resolve some of the issues mentioned above, we have devised a novel model-Hamiltonian method, the dynamical projective operatorial approach (DPOA) [52,67,68]. The DPOA proves to be able to analyze complicated *real* setups with several bands and unravel the photo injection mechanisms in ultra-fast regimes very affordably [52,53]. The DPOA uses projection matrices to obtain the time evolution of composite operators [69,70,71,72,73,74]; hence, in principle, it is capable of obtaining any out-of-equilibrium multi-time multi-particle quantities of interest. The DPOA is not restricted to any specific type of materials. Moreover, for instance, it can also handle strongly correlated systems [75]. However, this manuscript primarily focuses on DPOA’s applications to pumped semiconductors [52,76,77,78,79,80].

In this paper, we review our previous results [67,68], providing a clear understanding of how the DPOA should be applied to real systems. Using a simple model, we present the features that can be expected in pumped semiconductors, either via the measurement of the TR-ARPES signal or the transient optical properties. The rest of this manuscript is organized as follows: In Section 2, we present the theoretical framework of the DPOA. Section 2.1 introduces the general DPOA theory. In Section 2.2, the formalism is adapted to lattice systems. In Section 2.3, the formulation for computing the TR-ARPES signal is given. In Section 2.4, a generalized linear response theory for computing transient optical properties is reported. In Section 3, we consider a simplified modelization of a semiconductor and, through the application of theDPOA, we study its TR-ARPES signal and transient optical properties. Finally, in Section 4, we summarize and draft some conclusions.

## 2. Theory

### 2.1. Dynamical Projective Operatorial Approach (DPOA): General Theory

Consider a general time-independent Hamiltonian H which describes a system in equilibrium. It is always possible to find some sets of *composite* operators $C_{\alpha }^{†}=\left(C_{\alpha ,1}^{†},\ldots ,C_{\alpha ,a}^{†},\ldots \right)$, which close their hierarchy of the equations of motion (EOMs), where α identifies the set’s number [81,82,83,84,85,86,87,88,89,90,91,92,93,94,95,96]. The total number of (disjoint) sets is determined by the number of (independent—non-correlated) degrees of freedom characterizing the system [69,70,71,72,73,74].

In the presence of a time-dependent external perturbation (e.g., an electromagnetic pump pulse), the Hamiltonian becomes time-dependent: $H\to H\left(t\right)$. Such a perturbation usually affects only the the single-particle term of the Hamiltonian [66] and the *closure* of the hierarchy of the EOMs of the $C_{\alpha }$ is preserved, as follows:

(1) $$i\hbar \partial_{t}C_{\alpha }\left(t\right)=\left[C_{\alpha }\left(t\right),H\left(t\right)\right]=\Xi_{\alpha }\left(t\right)\cdot C_{\alpha }\left(t\right),$$

where · is the matrix product in the operatorial space, and $\Xi_{\alpha }\left(t\right)$ and $C_{\alpha }\left(t\right)$ are the time-dependent energy matrix and eigenoperators within the set α in the Heisenberg picture, respectively. The dynamical projective operatorial approach (DPOA) exploits that [67]

(2) $$C_{\alpha }\left(t\right)=P_{\alpha }\left(t,t_{\mathrm{ini}}\right)\cdot C_{\alpha }\left(t_{\mathrm{ini}}\right)\;\forall t\geq t_{\mathrm{ini}},$$

where $P_{\alpha }\left(t,t_{\mathrm{ini}}\right)$ is the dynamical projection matrix. In the following, we choose $t_{\mathrm{ini}}$ to be any time before the application of the pump pulse (e.g., $t_{\mathrm{ini}}\to -\infty$), so that $C_{\alpha }\left(t_{\mathrm{ini}}\right)$ stands for the operatorial basis describing the equilibrium system. Moreover, for the sake of simplicity, we select $P_{\alpha }\left(t,t_{\mathrm{ini}}\right)\to P_{\alpha }\left(t\right)$. The EOM for $P_{\alpha }\left(t\right)$ reads as [67]

(3) $$i\hbar \partial_{t}P_{\alpha }\left(t\right)=\Xi_{\alpha }\left(t\right)\cdot P_{\alpha }\left(t\right),$$

which should be solved with the initial condition $P_{\alpha }\left(t_{\mathrm{ini}}\right)=1$.

For a general pumped system, we have $\Xi_{\alpha }\left(t\right)=\Xi_{\alpha }^{\mathrm{eq}}+\Xi_{\alpha }^{\mathrm{pu}}\left(t\right)$, in which $\Xi_{\alpha }^{\mathrm{eq}}$ describes the system at equilibrium. At equilibrium, the solution of Equation (3) is $P_{\alpha }^{\mathrm{eq}}\left(t\right)=e^{-\frac{i}{\hbar }\left(t-t_{\mathrm{ini}}\right)\Xi_{\alpha }^{\mathrm{eq}}}$. It is worth noting that by rewriting $P_{\alpha }\left(t\right)=P_{\alpha }^{\mathrm{eq}}\left(t\right)\cdot P_{\alpha }^{\mathrm{int}}\left(t\right)=e^{-\frac{i}{\hbar }\left(t-t_{\mathrm{ini}}\right)\Xi_{\alpha }^{\mathrm{eq}}}\cdot P_{\alpha }^{\mathrm{int}}\left(t\right)$, we obtain the following reduced equation of motion:

(4) $$i\hbar \partial_{t}P_{\alpha }^{\mathrm{int}}\left(t\right)=\Xi_{\alpha }^{\mathrm{pu},\mathrm{int}}\left(t\right)\cdot P_{\alpha }^{\mathrm{int}}\left(t\right),$$

where $\Xi_{\alpha }^{\mathrm{pu},\mathrm{int}}\left(t\right)=e^{\frac{i}{\hbar }\left(t-t_{\mathrm{ini}}\right)\Xi_{\alpha }^{\mathrm{eq}}}\cdot \Xi_{\alpha }^{\mathrm{pu}}\left(t\right)\cdot e^{-\frac{i}{\hbar }\left(t-t_{\mathrm{ini}}\right)\Xi_{\alpha }^{\mathrm{eq}}}$. It is straightforward to show that, from Equation (4), one can obtain the following equivalent integro-differential equation:

(5) $$P_{\alpha }^{\mathrm{int}}\left(t\right)=1-\frac{i}{\hbar }\int_{t_{\mathrm{ini}}}^{t}dt^{\prime }\Xi_{\alpha }^{\mathrm{pu},\mathrm{int}}\left(t^{\prime }\right)\cdot P_{\alpha }^{\mathrm{int}}\left(t^{\prime }\right).$$

### 2.2. Pumped Lattice Systems out of Equilibrium

The DPOA can be applied to any kind of system: atoms, molecules, organic structures, lattice systems, etc. In this work, we focus on pumped lattice systems. Starting from the results of the many available DFT codes for the target equilibrium system and *wannierizing* them (for example, by exploiting the Wannier90 code [97]), one obtains a tight-binding quadratic Hamiltonian, which allows us to consider the effects of applying external EM fields (see, for instance, [52]). One should keep in mind that such an approach neglects the *dynamical* Coloumb interaction (i.e., its differential contribution to the out-of-equilibrium dynamics), even though it considers the *static* Coloumb interaction (through the exchange and correlation integrals within the equilibrium DFT calculation), which is fundamental for correctly describing the bands of the target system, for example, by opening and determining the value of the band gaps.

We consider an electromagnetic pump pulse with vector potential $A\left(t\right)$ and electric field $E\left(t\right)=-\partial_{t}A\left(t\right)$ applied to our system after some time $t_{\mathrm{ini}}$. In the dipole gauge, the related time-dependent Hamiltonian is given by

(6) $$H\left(t\right)=\sum_{k,\nu ,\nu^{\prime }}{\tilde{c}}_{k,\nu }^{†}\left(t\right){\tilde{\Xi }}_{k,\nu ,\nu^{\prime }}\left(t\right){\tilde{c}}_{k,\nu^{\prime }}\left(t\right),$$

where ${\tilde{c}}_{k,\nu }\left(t\right)$ is the electronic annihilation operator, k is the lattice momentum in the first Brillouin zone (FBZ), and ν, which also includes the spin, refers to the set of quantum numbers characterizing a localized state (e.g., a maximally localized Wannier state) and [66,67]

(7) $${\tilde{\Xi }}_{k,\nu ,\nu^{\prime }}\left(t\right)={\tilde{T}}_{k+\frac{e}{\hbar }A\left(t\right),\nu ,\nu^{\prime }}+eE\left(t\right)\cdot {\tilde{D}}_{k+\frac{e}{\hbar }A\left(t\right),\nu ,\nu^{\prime }}.$$

${\tilde{T}}_{k,\nu ,\nu^{\prime }}$ and ${\tilde{D}}_{k,\nu ,\nu^{\prime }}$ are the hopping and dipole matrix elements, respectively. We have adopted a notation in which any operator (in either first or second quantization) written in the basis of the localized states carries the over-script ∼. The value of the electronic charge is indicated by e>0. Moreover, ·, in this and similar cases, stands for the dot product between two vectors in the Cartesian space. Equation (7) can be considered as the generalization of the Peierls substitution [98,99] to multi-band systems [66].

It is convenient to work within a basis in which the equilibrium Hamiltonian, ${\tilde{\Xi }}_{k,\nu ,\nu^{\prime }}\left(t\leq t_{\mathrm{ini}}\right)={\tilde{T}}_{k,\nu ,\nu^{\prime }}$, is diagonal. The transformation to such a basis, which we call the band basis, can be performed through a unitary matrix $\Omega_{k,\nu ,n}$ satisfying

(8) $$\sum_{\nu ,\nu^{\prime }}\Omega_{k,n,\nu }^{†}{\tilde{T}}_{k,\nu ,\nu^{\prime }}\Omega_{k,\nu^{\prime },n^{\prime }}=\delta_{n,n^{\prime }}\varepsilon_{k,n},$$

where $\delta_{n,n^{\prime }}$ stands for the Kronecker delta, *n* is the index of the energy band and $\varepsilon_{k,n}$ is the band energy. Using $\Omega_{k,\nu ,n}$, it is straightforward to transform any matrix from the localized basis to the band basis:

(9) $$\begin{array}{c} M_{k,n,n^{\prime }}=\sum_{\nu ,\nu^{\prime }}\Omega_{k,n,\nu }^{†}{\tilde{M}}_{k,\nu ,\nu^{\prime }}\Omega_{k,\nu^{\prime },n^{\prime }}, \end{array}$$

where *M* can be any of *T*, D, Ξ, etc. In order to clearly distinguish the operators in the two bases, we denote the operators in the band basis without the over-script∼used for the operators in the localized basis. The annihilation operator of an electron in the band *n* and with momentum k is as follows:

(10) $$c_{k,n}\left(t\right)=\sum_{\nu }\Omega_{k,n,\nu }^{†}{\tilde{c}}_{k,\nu }\left(t\right).$$

Out of equilibrium, we have $c_{k}\left(t\right)=P_{k}\left(t\right)\cdot c_{k}\left(t_{\mathrm{ini}}\right)$, where $P_{k}\left(t_{\mathrm{ini}}\right)=1$ and $i\hbar \partial_{t}P_{k}\left(t\right)=\Xi_{k}\left(t\right)\cdot P_{k}\left(t\right)$. Moreover, the out-of-equilibrium number of electrons in band *n* with momentum k, $N_{k,n}\left(t\right)=\langle c_{k,n}^{†}\left(t\right)c_{k,n}\left(t\right)\rangle$, can be calculated as

(11) $$N_{k,n}\left(t\right)=\sum_{n^{\prime }}P_{k,n,n^{\prime }}\left(t\right)f_{k,n^{\prime }}P_{k,n^{\prime },n}^{†}\left(t\right),$$

where $f_{k,n}$ is the Fermi distribution function.

In *real* materials, one usually deals with several bands and a dense k grid: computing the out-of-equilibrium Hamiltonian terms (matrices with band indexes per each momentum value in the grid and at each instant of time) is extremely time-consuming. A very efficient way to noticeably reduce the computational cost is to expand any matrix $M_{k+\frac{e}{\hbar }A\left(t\right)}$ in powers of the vector potential [67]:

(12) $$\begin{array}{c} M_{k+\frac{e}{\hbar }A\left(t\right)}\left(t\right)=\sum_{m=0}^{\infty }\frac{1}{m!}\Omega_{k}^{†}\cdot \left[\partial_{k_{A}}^{\left(m\right)}{\tilde{M}}_{k}\right]\cdot \Omega_{k}{\left(\frac{e}{\hbar }A\left(t\right)\right)}^{m}. \end{array}$$

The vector potential is expressed as $A\left(t\right)=A\left(t\right)\hat{A}$, where $\hat{A}$ is the polarization of the pulse and $A\left(t\right)$ is its amplitude. $\partial_{k_{A}}^{\left(m\right)}$ is the *m*-th partial derivative in the direction of $\hat{A}$. Judiciously truncating the *Peierls expansion*, Equation (12), one needs to compute only once the properly chosen number of derivatives per each momentum value and use them at all times. The *m*-th partial derivatives can be computed as follows:

(13) $$\begin{array}{c} \partial_{k_{A}}^{\left(m\right)}{\tilde{M}}_{k}=\sum_{i}{\left(-i\hat{A}\cdot R_{i}\right)}^{m}e^{-ik\cdot R_{i}}{\tilde{M}}_{R_{i}}, \end{array}$$

where ${\tilde{M}}_{R_{i}}$ is the matrix in the direct space, as computed via the wannierization procedure. For the hopping matrix, M=T, we call the term $\Omega_{k}^{†}\cdot \left[\partial_{k_{A}}^{\left(m\right)}{\tilde{T}}_{k}\right]\cdot \Omega_{k}$ with m=1 (m=2) the velocity (inverse-mass) term. However, even though the derivative is not applied to the transformation matrix, $\Omega_{k}$, the resulting quantity is not the velocity (inverse mass) relative to the energy bands.

### 2.3. Green’s Functions and TR-ARPES Signal

Green’s function (GF) formalism provides a very versatile machinery to compute several relevant properties of a system. The retarded, $G^{R}$, and the lesser, $G^{<}$, GFs, are defined as follows:

(14) $$\begin{array}{cc} \; & G_{k,n,n^{\prime }}^{R}\left(t,t^{\prime }\right)=-i\theta \left(t-t^{\prime }\right)\langle \left\{c_{k,n}\left(t\right),c_{k,n^{\prime }}^{†}\left(t^{\prime }\right)\right\}\rangle , \end{array}$$

(15) $$\begin{array}{cc} \; & G_{k,n,n^{\prime }}^{<}\left(t,t^{\prime }\right)=i\langle c_{k,n^{\prime }}^{†}\left(t^{\prime }\right)c_{k,n}\left(t\right)\rangle . \end{array}$$

Using the DPOA, one can obtain non-equilibrium GFs in terms of the dynamical projection matrices *P* as

(16) $$\begin{array}{cc} \; & G_{k,n,n^{\prime }}^{R}\left(t,t^{\prime }\right)=-i\theta \left(t-t^{\prime }\right)\sum_{m}P_{k,n,m}\left(t\right)P_{k,n^{\prime },m}^{★}\left(t^{\prime }\right), \end{array}$$

(17) $$\begin{array}{cc} \; & G_{k,n,n^{\prime }}^{<}\left(t,t^{\prime }\right)=i\sum_{m,m^{\prime }}\left(\delta_{m,m^{\prime }}-\rho_{k,m,m^{\prime }}\left(t_{\mathrm{ini}}\right)\right)P_{k,n,m}\left(t\right)P_{k,n^{\prime },m^{\prime }}^{★}\left(t^{\prime }\right), \end{array}$$

where $\rho_{k,m,m^{\prime }}\left(t_{\mathrm{ini}}\right)$ is the single-particle density matrix at equilibrium. In the band basis in which the equilibrium Hamiltonian is diagonal, $\delta_{n,n^{\prime }}-\rho_{k,n,n^{\prime }}\left(t_{\mathrm{ini}}\right)=\delta_{n,n^{\prime }}f_{k,n}$.

For determining the out-of-equilibrium particle distribution over the energy of a pumped system, one can analyze the TR-ARPES signal [100,101,102,103], which plays almost the same role out of equilibrium of the spectral function in equilibrium. In this case, in addition to the pump pulse, one needs to consider a probe pulse too. The probe pulse ejects the electrons out of the system so that their energy and momentum can be measured: this is known as the TR-ARPES signal. This, for a probe pulse centered at $t_{\mathrm{pr}}$, is proportional to (see [67] and the references therein)

(18) $$\begin{array}{c} I_{k}^{<}\left(\omega ,t_{\mathrm{pr}}\right)=\frac{\tau_{\mathrm{pr}}}{\sqrt{8\pi \ln2}}\int_{-\infty }^{+\infty }dt_{1}\int_{-\infty }^{+\infty }dt_{2}S_{\mathrm{pr}}\left(t_{1}-t_{\mathrm{pr}}\right)\times \\ \;\;\;\;\;\;\;\;\;\;\;\;\;\;\;\;\;\;\;\;\;\;\;\;\;\;\;\;\;\;\;\;\;\;\;\;\;\;\;\;\;\;\;\;\;\;\;\;\;\;\;\;\;\;\;\;\;\;\;\;\;\;\;\times S_{\mathrm{pr}}\left(t_{2}-t_{\mathrm{pr}}\right)ℑ\left[e^{i\omega \left(t_{1}-t_{2}\right)}\mathrm{Tr}\left[G_{k}^{<}\left(t_{1},t_{2}\right)\right]\right], \end{array}$$

where $S_{\mathrm{pr}}\left(t-t_{\mathrm{pr}}\right)=\frac{2\sqrt{\ln2}}{\sqrt{\pi }\tau_{\mathrm{pr}}}e^{-4\ln2{\left(t-t_{\mathrm{pr}}\right)}^{2}/\tau_{\mathrm{pr}}^{2}}$ is the probe–pulse envelope. The normalization factor is chosen in such a way that $I^{<}\left(\omega ,t_{\mathrm{pr}}\right)$ is normalized to the total number of particles at momentum k,

(19) $$\int_{-\infty }^{+\infty }d\omega I_{k}^{<}\left(\omega ,t_{\mathrm{pr}}\right)=\sum_{n}N_{k,n}.$$

Although $I_{k}^{<}\left(\omega ,t_{\mathrm{pr}}\right)$ provides information about the occupied states, the out-of-equilibrium TR-ARPES *bands* are computed using a retarded signal defined as [67]

(20) $$\begin{array}{c} I_{k}^{R}\left(\omega ,t_{\mathrm{pr}}\right)=-\frac{\tau_{\mathrm{pr}}}{\sqrt{2\pi \ln2}}\int_{-\infty }^{+\infty }dt_{1}\int_{-\infty }^{+\infty }dt_{2}S_{\mathrm{pr}}\left(t_{1}-t_{\mathrm{pr}}\right)\times \\ \;\;\;\;\;\;\;\;\;\;\;\;\;\;\;\;\;\;\;\;\;\;\;\;\;\;\;\;\;\;\;\;\;\;\;\;\;\;\;\;\;\;\;\;\;\;\;\;\;\;\;\;\;\;\;\;\;\;\;\;\;\;\;\;\;\;\times S_{\mathrm{pr}}\left(t_{2}-t_{\mathrm{pr}}\right)ℑ\left[e^{i\omega \left(t_{1}-t_{2}\right)}\mathrm{Tr}\left[G_{k}^{R}\left(t_{1},t_{2}\right)\right]\right]. \end{array}$$

In the band basis, we have [67]

(21) $$\begin{array}{cc} I_{k}^{<}\left(\omega ,t_{\mathrm{pr}}\right)= & \sum_{n,n^{\prime }}L_{k,n;n^{\prime }}\left(\omega ,t_{\mathrm{pr}}\right)f_{k,n^{\prime }}, \end{array}$$

(22) $$\begin{array}{cc} I_{k}^{R}\left(\omega ,t_{\mathrm{pr}}\right)= & \sum_{n,n^{\prime }}L_{k,n;n^{\prime }}\left(\omega ,t_{\mathrm{pr}}\right), \end{array}$$

where

(23) $$L_{k,n;n^{\prime }}\left(\omega ,t_{\mathrm{pr}}\right)=\frac{\tau_{\mathrm{pr}}}{2\sqrt{2\pi \ln2}}{\left|\int_{-\infty }^{+\infty }dt_{1}S_{\mathrm{pr}}\left(t_{1}-t_{\mathrm{pr}}\right)e^{i\omega t_{1}}P_{k,nn^{\prime }}\left(t_{1}\right)\right|}^{2},$$

which shows that the TR-ARPES signal is non-negative. It is worth noting that Equations (21) and (22) act as the out-of-equilibrium counterpart of the fluctuation–dissipation theorem.

### 2.4. Out-of-Equilibrium Optical Properties

In studying the out-of-equilibrium optical properties of a pumped system, we need to consider an overall electromagnetic pulse composed of both a pump and a probe pulse. The system’s optical response (reflection and/or absorption) is generally studied as a function of the time delay between the pump and probe pulses. Analyzing such a variation, one obtains information about the ultra-fast pump-induced effects on the system [9,10,20,39,40,41,42,43,47,49,50,52,53,54].

Since the probe pulse is very weak by definition, one can compute the transient optical properties of the system through a generalized linear response theory (GLRT) [68] that fully takes into account that the system is under the effect of the pump pulse. Deriving such a GLRT requires the following [68]: (i) to obtain the light-matter Hamiltonian for a general realistic lattice system and a general electromagnetic pulse; (ii) to derive the expression of the electric current for such a pumped system; (iii) to determine the expression of the optical conductivity of a pumped system in this framework; (iv) to obtain the expressions of the transient reflectivity and absorption; (v) to devise a numerical framework that effectively permits us to compute these quantities for *real* materials (many conduction and valence bands involved and very dense momentum grids).

Within this framework, exploiting the DPOA, the out-of-equilibrium time-dependent optical conductivity of a pumped system probed at time $t_{\mathrm{pr}}$ (i.e., the center of the probe pulse is at time $t_{\mathrm{pr}}$), $\sigma \left(t,t_{\mathrm{pr}}\right)$, reads as follows: [68]

(24) $$\sigma \left(t,t_{\mathrm{pr}}\right)=\sigma_{1}\left(t,t_{\mathrm{pr}}\right)+\sigma_{2}\left(t,t_{\mathrm{pr}}\right),$$

where

(25) $$\begin{array}{c} \sigma_{1}\left(t,t_{\mathrm{pr}}\right)=\frac{ie}{\hbar V}\theta \left(t-t_{\mathrm{pr}}\right)\times \\ \;\;\;\;\;\;\;\;\;\;\times \sum_{k}\sum_{n_{1}n_{2}n_{3}n_{4}}\sum_{n_{1}^{\prime }n_{2}^{\prime }}J_{k,n_{1}n_{2}}\left(t\right)\int_{t_{\mathrm{ini}}}^{t}dt^{\prime }\left(-\frac{e}{\hbar }V_{k,n_{3}n_{4}}\left(t^{\prime }\right)\theta \left(t^{\prime }-t_{\mathrm{pr}}\right)+eD_{k,n_{3}n_{4}}\left(t^{\prime }\right)\delta \left(t^{\prime }-t_{\mathrm{pr}}\right)\right)\\ \;\;\;\;\;\;\;\;\;\;\;\;\;\;\;\;\;\;\;\;\;\;\;\;\;\;\;\;\;\;\;\;\;\;\;\;\;\;\;\;\;\;\;\;\;\;\;\;\;\;\;\;\;\;\;\;\;\;\;\;\;\;\;\;\;\;\;\;\;\;\;\;\;\;\;\;\;\;\;\;\;\;\;\;\;\;\;\;\;\;\;\;\;\;\;\;\;\;\;\;\;\;\;\;\;\;\;\times P_{kn_{1}n_{1}^{\prime }}^{★}\left(t\right)P_{kn_{2}n_{2}^{\prime }}\left(t\right)P_{kn_{3}n_{2}^{\prime }}^{★}\left(t^{\prime }\right)P_{kn_{4}n_{1}^{\prime }}\left(t^{\prime }\right)\left(f_{kn_{1}^{\prime }}-f_{kn_{2}^{\prime }}\right). \end{array}$$

which can be rewritten in a computationally more efficient way as follows:

(26) $$\sigma_{1}\left(t,t_{\mathrm{pr}}\right)=\frac{ie}{\hbar v_{\mathrm{uc}}N_{\mathrm{grid}}}\theta \left(t-t_{\mathrm{pr}}\right)\sum_{k\in \mathrm{grid}}\mathrm{Tr}\left\{Z_{k}\left(t\right)\cdot \left[Y_{k}\left(t\right)-Y_{k}\left(t_{\mathrm{pr}}\right)+X_{k}\left(t_{\mathrm{pr}}\right)\right]\right\},$$

where $N_{\mathrm{grid}}$ is the number of points on the grid which samples the FBZ, and $v_{\mathrm{uc}}$ is the volume of unit cell. Thus, the full volume of the system is $V=v_{\mathrm{uc}}N_{\mathrm{grid}}$. The matrices $Z_{k}\left(t\right)$, $Y_{k}\left(t\right)$ and $X_{k}\left(t\right)$ read as follows:

(27) $$Z_{k,n_{1}^{\prime }n_{2}^{\prime }}\left(t\right)=\sum_{n_{1}n_{2}}J_{k,n_{1}n_{2}}\left(t\right)P_{k,n_{1}n_{1}^{\prime }}^{★}\left(t\right)P_{k,n_{2}n_{2}^{\prime }}\left(t\right)\left(f_{kn_{1}^{\prime }}-f_{kn_{2}^{\prime }}\right),$$

(28) $$Y_{k,n_{2}^{\prime }n_{1}^{\prime }}\left(t\right)=-\frac{e}{\hbar }\sum_{n_{3}n_{4}}\int_{t_{\mathrm{ini}}}^{t}dt^{\prime }V_{k,n_{3}n_{4}}\left(t^{\prime }\right)P_{k,n_{3}n_{2}^{\prime }}^{★}\left(t^{\prime }\right)P_{k,n_{4}n_{1}^{\prime }}\left(t^{\prime }\right),$$

(29) $$X_{k,n_{2}^{\prime }n_{1}^{\prime }}\left(t\right)=e\sum_{n_{3}n_{4}}D_{k,n_{3}n_{4}}\left(t\right)P_{k,n_{3}n_{2}^{\prime }}^{★}\left(t\right)P_{k,n_{4}n_{1}^{\prime }}\left(t\right),$$

and

(30) $$J_{k,n_{1}n_{2}}\left(t\right)=\frac{1}{\hbar }\eta_{k,n_{1}n_{2}}\left(t\right)-\frac{i}{\hbar }{\left[D_{k}\left(t\right),T_{k}\left(t\right)\right]}_{n_{1}n_{2}},$$

(31) $$\eta_{k,n_{1}n_{2}}\left(t\right)=\sum_{\nu_{1}\nu_{2}}\Omega_{k\nu_{1}n_{1}}^{*}\left(\nabla_{k}{\tilde{T}}_{k+\frac{e}{\hbar }A_{\mathrm{pu}}\left(t\right),\nu_{1}\nu_{2}}\right)\Omega_{k\nu_{2}n_{2}},$$

(32) $$V_{k,n_{1}n_{2}}\left(t\right)=\eta_{k,n_{1}n_{2}}\left(t\right)+e\Lambda_{k,n_{1}n_{2}}\left(t\right)\cdot E_{\mathrm{pu}}\left(t\right),$$

(33) $$\Lambda_{k,n_{1}n_{2}}\left(t\right)=\sum_{\nu_{1}\nu_{2}}\Omega_{k\nu_{1}n_{1}}^{*}\left(\nabla_{k}{\tilde{D}}_{k+\frac{e}{\hbar }A_{\mathrm{pu}}\left(t\right),\nu_{1}\nu_{2}}\right)\Omega_{k\nu_{2}n_{2}},$$

where the notation ${\left[\Phi ,\Psi \right]}_{n_{1}n_{2}}=\sum_{n^{\prime }}\left(\Phi_{n_{1}n^{\prime }}\Psi_{n^{\prime }n_{2}}-\Psi_{n_{1}n^{\prime }}\Phi_{n^{\prime }n_{2}}\right)$ is used for the commutator.

Moreover [68],

(34) $$\sigma_{2}\left(t,t_{\mathrm{pr}}\right)=\frac{e}{V}\theta \left(t-t_{\mathrm{pr}}\right)\sum_{n_{1}n_{2}k}\frac{\delta J_{k,n_{1}n_{2}}\left(t\right)}{\delta A\left(t\right)}N_{kn_{1}n_{2}}\left(t\right),$$

where

(35) $$N_{kn_{1}n_{2}}\left(t\right)=\sum_{n^{\prime }}P_{kn_{1}n^{\prime }}^{★}\left(t\right)P_{kn_{2}n^{\prime }}\left(t\right)f_{kn^{\prime }},$$

and

(36) $$\frac{\delta J_{k,n_{1}n_{2}}\left(t\right)}{\delta A\left(t\right)}=\frac{e}{\hbar^{2}}\xi_{k,n_{1}n_{2}}\left(t\right)-\frac{ie}{\hbar^{2}}{\left[\Lambda_{k}\left(t\right),T_{k}\left(t\right)\right]}_{n_{1}n_{2}}-\frac{ie}{\hbar^{2}}{\left[D_{k}\left(t\right),\eta_{k}\left(t\right)\right]}_{n_{1}n_{2}},$$

in which

(37) $$\xi_{k,n_{1}n_{2}}\left(t\right)=\sum_{\nu_{1}\nu_{2}}\Omega_{k\nu_{1}n_{1}}^{*}\left[\nabla_{k}^{2}{\tilde{T}}_{k+\frac{e}{\hbar }A_{\mathrm{pu}}\left(t\right),\nu_{1}\nu_{2}}\right]\Omega_{k\nu_{2}n_{2}}.$$

Having the optical conductivity $\sigma \left(t,t_{\mathrm{pr}}\right)$, one performs the Fourier transformation with respect to $t-t_{\mathrm{pr}}$ to obtain it as a function of the probe–pulse frequency, ω, as

(38) $$\sigma \left(\omega ,t_{\mathrm{pr}}\right)=\int_{-\infty }^{+\infty }e^{i\left(\omega +i0^{+}\right)\left(t-t_{\mathrm{pr}}\right)}\sigma \left(t,t_{\mathrm{pr}}\right)dt,$$

where $0^{+}$ is a damping factor. In real systems, this damping factor is finite. In numerical simulations, one needs to choose its value while considering the specific system under study and the details of the simulation (see ref. [68] for a detailed discussion on this issue). However, to study pump-induced dynamics, the system should have dampings small enough such that the damping time ($∼2\pi /0^{+}$) is much larger than the time scales of those pump-induced dynamics.

A noticeable simplification in the calculations is achieved if one restricts the upper limit of the integral given in Equation (38) to some $t_{\mathrm{fin}}>t_{\mathrm{pr}}$ at which the pump pulse is negligible and writes

(39) $$\sigma \left(\omega ,t_{\mathrm{pr}}\right)=\int_{-\infty }^{t_{\mathrm{fin}}}e^{i\left(\omega +i0^{+}\right)\left(t-t_{\mathrm{pr}}\right)}\sigma \left(t,t_{\mathrm{pr}}\right)dt+\sigma^{a.p.}\left(\omega ,t_{\mathrm{pr}}\right),$$

where $\sigma^{a.p.}\left(\omega ,t_{\mathrm{pr}}\right)=\sigma_{1}^{a.p.}\left(\omega ,t_{\mathrm{pr}}\right)+\sigma_{2}^{a.p.}\left(\omega ,t_{\mathrm{pr}}\right)$ in which [68]

(40) $$\sigma_{1}^{a.p.}\left(\omega ,t_{\mathrm{pr}}\right)=-\frac{ie}{\hbar V}\sum_{k}\left\{W_{k}\left(\omega ,t_{\mathrm{fin}},t_{\mathrm{pr}}\right)+\mathrm{Tr}\left[Q_{k}\left(\omega ,t_{\mathrm{fin}},t_{\mathrm{pr}}\right)\cdot S_{k}\left(t_{\mathrm{fin}},t_{\mathrm{pr}}\right)\right]\right\},$$

where

(41) $$\begin{array}{c} Q_{k,n^{\prime }n}\left(\omega ,t_{\mathrm{fin}},t_{\mathrm{pr}}\right)=\sum_{n_{1}n_{2}}P_{k,n_{2}n^{\prime }}^{*}\left(t_{\mathrm{fin}}\right)J_{k,n_{2}n_{1}}P_{k,n_{1}n}\left(t_{\mathrm{fin}}\right)\times \\ \;\;\;\;\;\;\;\;\;\;\;\;\;\;\;\;\;\;\;\;\;\;\;\;\;\;\;\;\;\;\;\;\;\;\;\;\;\;\;\;\;\;\;\;\;\;\;\;\;\;\;\;\;\;\;\;\;\;\;\;\;\;\;\;\;\;\;\;\;\;\;\;\;\;\;\;\;\;\;\times \frac{ie^{\left(i\omega -0^{+}\right)\left(t_{\mathrm{fin}}-t_{\mathrm{pr}}\right)}}{\left(\omega -\omega_{k,n_{1}n_{2}}+i0^{+}\right)}\left(f_{kn^{\prime }}-f_{kn}\right), \end{array}$$

(42) $$S_{k}\left(t_{\mathrm{fin}},t_{\mathrm{pr}}\right)=-Y_{k}\left(t_{\mathrm{fin}}\right)+Y_{k}\left(t_{\mathrm{pr}}\right)-X_{k}\left(t_{\mathrm{pr}}\right),$$

(43) $$\begin{array}{c} W_{k}\left(\omega ,t_{\mathrm{fin}},t_{\mathrm{pr}}\right)=-\frac{e}{\hbar }\sum_{n_{1}n_{2}}\frac{e^{\left(i\omega -0^{+}\right)\left(t_{\mathrm{fin}}-t_{\mathrm{pr}}\right)}J_{k,n_{2}n_{1}}}{\omega -\omega_{k,n_{1}n_{2}}+i0^{+}}\times \\ \;\;\;\;\;\;\;\;\;\;\;\;\;\;\;\;\;\;\;\;\;\;\;\times \sum_{n}\left(\frac{\eta_{k,n_{1}n}}{\omega -\omega_{k,nn_{2}}+i0^{+}}N_{k,n_{2}n}\left(t_{\mathrm{fin}}\right)-\frac{\eta_{k,nn_{2}}}{\omega -\omega_{k,n_{1}n}+i0^{+}}N_{k,nn_{1}}\left(t_{\mathrm{fin}}\right)\right), \end{array}$$

and [68]

(44) $$\sigma_{2}^{a.p.}\left(\omega ,t_{\mathrm{pr}}\right)=\frac{e}{V}\sum_{k}\sum_{n_{1}n_{2}}\frac{iN_{k,n_{1}n_{2}}\left(t_{\mathrm{fin}}\right)e^{\left(i\omega -0^{+}\right)\left(t_{\mathrm{fin}}-t_{\mathrm{pr}}\right)}\frac{\delta J_{k.n_{1}n_{2}}}{\delta A}}{\omega_{k,n_{1}n_{2}}+\omega +i0^{+}}.$$

We consider a case in which both the pump and probe pulses are linearly polarized and both of them have the same polarization. Therefore, the optical conductivity reduces to $\sigma \left(\omega ,t_{\mathrm{pr}}\right)=\hat{A}\cdot \sigma \left(\omega ,t_{\mathrm{pr}}\right)\cdot \hat{A}$. Correspondingly, the dielectric function is

(45) $$\epsilon \left(\omega ,t_{\mathrm{pr}}\right)=1+\frac{i}{\omega \epsilon_{0}}\sigma \left(\omega ,t_{\mathrm{pr}}\right),$$

where $\epsilon_{0}$ is the vacuum dielectric constant.

If the probe pulse frequencies are much larger than the pump pulse ones, the transient reflectivity of an s-polarized probe pulse of central frequency ω and incident angle θ is given by

(46) $$R_{\theta }\left(\omega ,t_{\mathrm{pr}}\right)={\left|\frac{\cos\theta -\sqrt{\epsilon \left(\omega ,t_{\mathrm{pr}}\right)-\sin^{2}\theta }}{\cos\theta +\sqrt{\epsilon \left(\omega ,t_{\mathrm{pr}}\right)-\sin^{2}\theta }}\right|}^{2}.$$

Following the experimental measurements, we also compute the relative differential transient reflectivity, $\delta_{r}R_{\theta }\left(\omega ,t_{\mathrm{pr}}\right)$, defined as [52]

(47) $$\delta_{r}R_{\theta }\left(\omega ,t_{\mathrm{pr}}\right)=\frac{R_{\theta }\left(\omega ,t_{\mathrm{pr}}\right)-R_{\theta }^{\mathrm{eq}}\left(\omega \right)}{R_{\theta }^{\mathrm{eq}}\left(\omega \right)},$$

where $R_{\theta }^{\mathrm{eq}}\left(\omega \right)$ is the equilibrium reflectivity. To gain more information about the features appearing in $\delta_{r}R_{\theta }\left(\omega ,t_{\mathrm{pr}}\right)$, one can perform a Fourier transformation with respect to the time $t_{\mathrm{pr}}$:

(48) $$\delta_{r}R_{\theta }\left(\omega ,\omega^{\prime }\right)=\int_{-\infty }^{+\infty }e^{i\omega t_{\mathrm{pr}}}\delta_{r}R_{\theta }\left(\omega ,t_{\mathrm{pr}}\right)dt_{\mathrm{pr}}.$$

Up until now, we have been considering reflectivity. However, it is also possible to study the transient absorption coefficient of the pumped system, which is given by

(49) $$\alpha \left(\omega ,t_{\mathrm{pr}}\right)=\frac{\omega }{n_{\mathrm{refr}}\left(\omega ,t_{\mathrm{pr}}\right)c}ℑ\left[\epsilon \left(\omega ,t_{\mathrm{pr}}\right)\right],$$

where *c* is the speed of light in a vacuum and $n_{\mathrm{refr}}\left(\omega ,t_{\mathrm{pr}}\right)=ℜ\left[\sqrt{\epsilon \left(\omega ,t_{\mathrm{pr}}\right)}\right]$ is the real out-of-equilibrium refractive index. In our calculations, we compute the transient differential absorption coefficient as

(50) $$\delta \alpha \left(\omega ,t_{\mathrm{pr}}\right)=\alpha \left(\omega ,t_{\mathrm{pr}}\right)-\alpha^{\mathrm{eq}}\left(\omega \right),$$

where $\alpha^{\mathrm{eq}}\left(\omega \right)$ stands for the absorption coefficient at equilibrium.

## 3. The Case Study of a Three-Band Semiconductor

In both cases, the TR-ARPES signal and transient optical properties, to analyze the phenomenology that emerges in a pump–probe setup, it is helpful to consider a simple model describing a semiconductor. In this way, without becoming lost in the complications of any particular *real* material, one can construct a dictionary of phenomena coming from different mechanisms. Dealing with *real* experimental setups, such a dictionary can be extremely useful in analyzing the results.

Our semiconductor model consists of a cubic lattice with a lattice constant *a* and three bands: a valence band (VB), a conduction band (CB), and a *core* band. The dispersions of VB and CB can be obtained by the following hopping matrix elements: ${\tilde{T}}_{0,1,1}=-1.65\Delta$, ${\tilde{T}}_{0,2,2}=1.35\Delta$, ${\tilde{T}}_{a,1,1}=0.2\Delta$, ${\tilde{T}}_{a,2,2}=-0.15\Delta$, and ${\tilde{T}}_{a,1,2}={\tilde{T}}_{a,2,1}=-0.1\Delta$. Here, ${\tilde{T}}_{R,\nu ,\nu^{\prime }}$ is the hopping matrix element between localized Wannier states with indices ν and $\nu^{\prime }$ centered at a relative distance R, and $a\in \left\{a\left(\pm 1,0,0\right),a\left(0,\pm 1,0\right),a\left(0,0,\pm 1\right)\right\}$. The unit of energy is Δ, which determines the band gap. For example, Δ=0.5eV results in a band gap of 0.75eV at Γ. By diagonalizing the matrix $\tilde{T}$, one obtains the dispersions of VB and CBs. On the other hand, to simplify the model and focus on the effects of the pump pulse and not on the specific features of the *core* band and of its coupling to VB and CB, we consider a flat *core* band at energy $\varepsilon_{\mathrm{core}}=-50\Delta$ and no hopping between the *core* band and the other two bands. The *core* band is coupled to the VB and CB via a local momentum-independent dipole matrix element $D=i0.05a\hat{j}$. For the case of the TR-ARPES signal, we consider the *main* cubic path in the FBZ, while for studying the transient optical properties, we sample the **k** space by a cubic 32×32×32 grid, which includes Γ. The damping factor is chosen as $0^{+}=0.05\Delta /\hbar$.

The vector potential of the pump pulse is $A_{\mathrm{pu}}\left(t\right)=A_{\mathrm{pu}}\left(t\right)\hat{j}$ where $A_{\mathrm{pu}}\left(t\right)$ is

(51) $$A_{\mathrm{pu}}\left(t\right)=A_{0}e^{-\left(4\ln2\right)t^{2}/\tau_{\mathrm{pu}}^{2}}\cos\left(\omega_{\mathrm{pu}}t\right).$$

Accordingly, we assume that the center of the pump pulse is taken as the origin of the time axis. Hence, the center of the probe pulse, $t_{\mathrm{pr}}$, coincides with the time delay between the pump and probe pulses. The pump amplitude is $A_{0}=0.4\pi \hbar /ae$, the FWHM of the pump pulse is $\tau_{\mathrm{pu}}=7\hbar /\Delta$, and the pump frequency is $\omega_{\mathrm{pu}}=2.33\Delta /\hbar$.

### 3.1. TR-ARPES Signals

**Figure 1:**
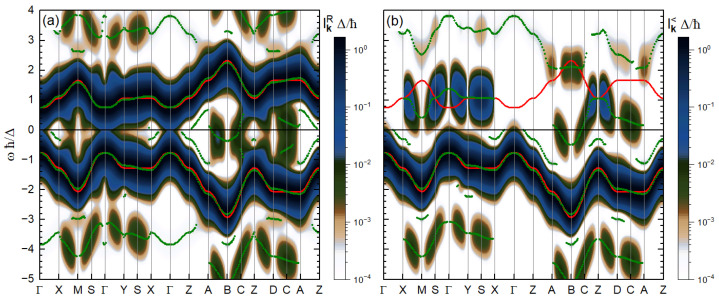
(**a**) The retarded, $I_{k}^{R}$, and (**b**) the lesser, $I_{k}^{<}$, TR-ARPES signals along the *main* path, for the case where the centers and the FWHM of the pump and probe pulses coincide with each other (i.e., $t_{\mathrm{pr}}=0$ and $\tau_{\mathrm{pr}}=\tau_{\mathrm{pu}}$). The equilibrium band energies are shown with solid red lines, and the local maxima of the signal at each **k** point are shown with green dots. The latter indicate the out-of-equilibrium TR-ARPES bands.

In Figure 1, we show the maps of the retarded and lesser TR-ARPES signals, when the center and the FWHM of the probe pulse coincides with those of the pump pulse. The local maxima of the TR-ARPES signal can be considered as the TR-ARPES *bands*. In the proximity of the equilibrium VB and CB (shown by the red lines), we have the main TR-ARPES *bands* that are just slightly shifted. Meanwhile, in equilibrium, we only have two bands in the energy range of Figure 1, where, under the application of the pump pulse, new side bands emerge. Some of these sidebands are∼$n\hbar \omega_{\mathrm{pu}}$ (*n* being a non-zero integer), apart from the main VB or CB, and we call them photon sidebands (PSBs), or more precisely, *n*-photon PSBs. We also witness the emergence of some other kinds of sidebands in the proximity of the main bands, which we called envelope-Peierls sidebands (EPSBs) [67]. The retarded signal features both VB and CB and all their sidebands, regardless of their occupation. On the other hand, the lesser signal, which is directly measurable in experiments, just shows the occupied portion of the band structure. Hence, it features only the VB and its sidebands.

The origin of the one-photon PSBs is the velocity term in the *Peierls expansion*. One can simply show that this velocity is proportional to $\sin\left(ak_{y}\right)$ in our model, which vanishes on the planes Γ-**X**-**A**-**Z** and **Y**-**M**-**B**-**D**, preventing the appearance of one-photon PSBs at these **k**-points. The inverse-mass term is proportional to $\cos\left(ak_{y}\right)$. Therefore, at **S**, **C**, middle points of the lines **X**-**M**, **A**-**B**, **Z**-**D** and Γ-**Y**, it vanishes identically. The vanishing of inverse-mass terms results in no shift of the TR-ARPES bands with respect to the equilibrium bands, but it results in the disappearance of the EPSBs. This clarifies how the symmetries of the system can affect the emerging features in the TR-ARPES signals, namely the band shifts and the pump-induced sidebands.

### 3.2. Transient Optical Properties

**Figure 2:**
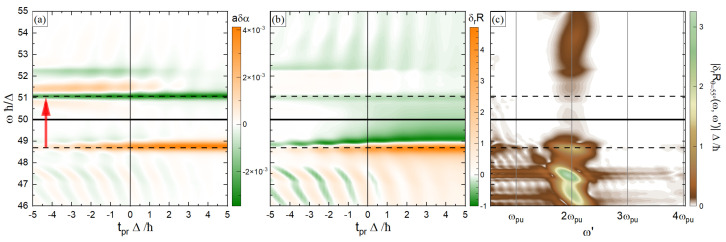
(**a**) Differential absorption coefficient (Equation (50)), as a function of $t_{\mathrm{pr}}$ and ω. The absorption coefficient is made to be dimensionless by being multiplied by the lattice constant *a*. The red arrow shows the electronic excitation via the one-photon resonance with the pump pulse. (**b**) Relative differential reflectivity for θ=55° (Equation (47)) as a function of $t_{\mathrm{pr}}$ and ω. (**c**) The frequency content of relative differential reflectivity, $\left|\delta_{r}R_{\theta =55{}^{\circ}}\left(\omega ,\omega^{\prime }\right)\right|$ (see Equation (48)). The low-frequency region, $\omega^{\prime }<0.2\Delta /\hbar$, is not shown.

In Figure 2, the transient optical properties of the system under the application of the pump pulse are shown. The low-frequency pump pulse excites the electrons from the VB to the CB, while a high-frequency probe pulse, which is in the frequency range of the energy gaps between the *core* band and VB/CB, allows us to detect the effects of the pump pulse in transient optical properties.

Figure 2a reports the differential absorption coefficient, as shown in Equation (50). The one-photon resonances between the VB and CB mainly occur at the indicated dashed lines, where the absorption coefficient shows its most significant changes. This can be understood as follows: The pump pulse strongly excites electrons from the VB to the CB at resonant k points, and it creates holes in the former and electrons in the latter. The holes in the VB make it possible for the high-frequency probe pulse to excite electrons from the *core* band to the VB, which results in an increase in the absorption coefficient. On the other hand, because of the Pauli exclusion principle, the electrons in the CB leave less vacancies for the probe-induced excitations from the *core* band. Consequently, we have a reduction in the absorption coefficient at the corresponding probe frequencies.

Figure 2b reports the relative differential reflectivity at the angle *θ* = 55° (see Equation (47)). At the absorption edges, where the absorption coefficient undergoes the most significant changes (indicated with dashed lines), $\delta_{r}R$ approaches zero. Therefore, we obtain very narrow white strips. This can be very helpful in finding the connection between the experimental results for $\delta_{r}R$ and the band structure of the system.

Figure 2c reports $\left|\delta_{r}R_{\theta =55{}^{\circ}}\left(\omega ,\omega^{\prime }\right)\right|$, which is the frequency content of $\delta_{r}R_{\theta =55{}^{\circ}}$(see Equation (48)). The oscillations of the relative differential reflectivity (in $t_{\mathrm{pr}}$, with respect to which the Fourier transformation is performed) are in the proximity of twice the pump frequency, $\omega^{\prime }=2\omega_{\mathrm{pu}}$, and there is no odd component. This shows that summing over all **k** points in the FBZ cancels out the odd frequencies that originate from the velocities.

## 4. Summary and Perspectives

In this manuscript, we reviewed our recently developed model-Hamiltonian approach, the dynamical projective operatorial approach (DPOA) [52,53,67,68]. The main ingredients of the DPOA and its applications to pumped semiconductors are reported and explained in detail. After providing the general theory of the DPOA, we clarified how it can exploit the equilibrium *ab initio* results for investigating pumped semiconductors through the study of the TR-ARPES signal and of the transient optical properties.

For the case of the TR-ARPES signal, we presented the methodology to compute the standard experimentally measurable signal, the lesser signal. Moreover, we computed another (theoretical) TR-ARPES signal, the retarded one, which can be used to study the out-of-equilibrium bands of pumped systems. In the case of transient optical properties, the results of our generalized linear response theory are presented and used to analyze a pumped system.

The DPOA can be applied to *real* materials (with [52] being an example). However, to illustrate different kinds of phenomena that can emerge in such pump–probe setups, we presented a simple yet rich model for a semiconductor. For the TR-ARPES signal, we discussed the emergence of different kinds of sidebands, the roles of system symmetries, and the relevance of the resonances. For the case of transient optical properties, we established the relations between the system band structure, the pump-induced excitations, the transient optical response of the system to the probe pulse, and the frequency content of absorption and reflectivity.

The DPOA is a versatile approach that can be applied to highly complicated systems with an affordable computational cost.

## Data Availability

The raw data supporting the conclusions of this article will be made available by the authors on request.
